# Osteoporotic Hip Fractures: The Burden of Fixation Failure

**DOI:** 10.1155/2013/515197

**Published:** 2013-02-06

**Authors:** J. M. Broderick, R. Bruce-Brand, E. Stanley, K. J. Mulhall

**Affiliations:** ^1^Mater Misericordiae University Hospital, Dublin, Ireland; ^2^Sports Surgery Clinic, Dublin, Ireland; ^3^Cappagh National Orthopaedic Hospital, Dublin, Ireland

## Abstract

Osteoporotic hip fractures are a major cause of morbidity and mortality in the elderly. Furthermore, reduced implant anchorage in osteoporotic bone predisposes towards fixation failure and with an ageing population, even low failure rates represent a significant challenge to healthcare systems. Fixation failure in fragility fractures of the hip ranges from 5% in peritrochanteric fractures through to 15% and 41% in undisplaced and displaced fractures of the femoral neck, respectively. Our findings, in general, support the view that failed internal fixation of these fragility fractures carries a poor prognosis: it leads to a twofold increase in the length of hospital stay and a doubling of healthcare costs. Patients are more likely to suffer a downgrade in their residential status upon discharge with a consequent increase in social dependency. Furthermore, the marked disability and reduction in quality of life evident before salvage procedures may persist at long-term followup. The risk, of course, for the elderly patient with a prolonged period of decreased functioning is that the disability becomes permanent. Despite this, however, no clear link between revision surgery and an increase in mortality has been demonstrated in the literature.

## 1. Introduction

Osteoporotic hip fractures are a substantial cause of morbidity and mortality in the elderly. That osteoporosis leads to fragility fractures is well known; however, underlying changes in bone mass and microarchitecture that manifest as fracture in the acute setting can also undermine subsequent attempts at fracture healing. Reduced implant anchorage in osteoporotic bone predisposes towards a failure of fixation [1] and with current projections estimating a worldwide increase in hip fractures from 1.7 million in 1990 to 6.3 million by 2050 [2], even low failure rates represent a significant challenge to healthcare systems. We have reviewed the incidence, the impact, and the additional economic cost of such fixation failures in osteoporotic fractures of the hip.

## 2. Review Methods

### 2.1. Defining Osteoporotic Fractures

The classic fragility fractures are those of the proximal femur, proximal humerus, distal radius, and vertebral fractures. There are, however, problems in determining what actually constitutes an osteoporotic fracture. The World Health Organization (WHO) defines osteoporosis as a bone mineral density (BMD) of 2.5 standard deviations (SDs) or more below the young normal mean [3]. However, this definition applies only to postmenopausal women assessed by dual-energy X-ray absorptiometry (DEXA) scan and no such definition exists for men. Previous attempts to estimate the degree of osteoporosis include the Singh index, which involves fitting the pattern of proximal femoral trabecular lines into six separate categories; however, this has been shown to have poor inter- and intraobserver reliability [4] and, moreover, it does not correlate with BMD as measured by DEXA scanning. Perhaps the most useful description of fragility fractures is that recently proposed by Kanis et al. [5] in defining osteoporotic fractures as those that occur at a site associated with low BMD and that increase in incidence over the age of 50 years. Indeed, hip fractures alone are regularly used as surrogate markers in determining the international burden of osteoporosis. 

### 2.2. Determinants of the Incidence of Fixation Failure

With multiple fixation devices available and most studies concentrating on one implant versus another, we have included all fixation types in our review. Only studies reporting on patients aged 60 years and older were included. 

Fixation failure was defined as

- nonunion (alternatively referred to in some studies as early, progressive, or redisplacement of the fracture or fracture collapse),
- avascular necrosis of the femoral head,
- cutout of the implant,
- implant penetration of the femoral head,
- “Z-effect”,
- breakage of the implant,
- detachment of the implant from the femur,
- intraoperative fracture of the femur and,
- later fracture of the femur.

Only excessive displacement necessitating revision surgery was classified as a fixation failure. Simple migration of the lag screw within the femoral head or a fracture that was noted to have united in “acceptable” varus, valgus, or rotation at routine followup was not classified as a fixation failure. Deep sepsis, despite being an important cause of patient morbidity and failed surgery, also fell outside our search criteria. 

Where the number of patients providing data for any outcome was reported, we used these provided data. In studies in which the denominator was unclear, we used the numbers randomised or alive at followup. 

### 2.3. Determinants of the Impact and Cost of Fixation Failure

We attempted to identify studies which specifically recorded final outcome measures in osteoporotic hip fracture patients who suffered a failed fixation. Data was extracted with regard to social and economic implications, namely, length of hospital stay, return to prefracture residential status, quality of life, and functioning as well as economic cost.

## 3. Results

### 3.1. Incidence of Fixation Failure

Undisplaced femoral neck fractures managed by internal fixation (IF) demonstrate an overall failure rate of 14.8% and a reoperation rate of 15.4% (Table 1). 

A significantly higher failure rate, however, occurs in displaced fractures of the femoral neck. Randomized controlled trials (RCTs) demonstrate that, overall, a failure rate of 41% and reoperation rate of 45.4% can be expected (Table 2). 

With regard to peritrochanteric fractures of the femur, the results of RCTs demonstrate a failure rate of 5% and reoperation rate of 4.9% (Table 3). Failure rates will vary, however, depending on the stability of the fracture type. While most studies included both stable and unstable fracture patterns, Varela-Egocheaga [6] solely focused on stable fracture types noting a failure rate of 3.8%; this can be contrasted with the findings of Sadowski et al. [7] who investigated only the most unstable fracture patterns (reverse oblique and transverse intertrochanteric) and noted a significant increase in failure rate to 22.9%. 

### 3.2. Impact of Fixation Failure

A hip fracture is probably the most devastating consequence of osteoporosis in our ageing population [8], with the concomitant increase in morbidity and mortality being well documented. Of particular interest, however, is the additional effect any fixation failure will have in this already frail population. 

#### 3.2.1. Length of Stay

On average, the failure of fixation results in a twofold increase in the length of hospital stay (Table 4). Thakar et al. [9] divided total hospital stay into acute—and community—stay periods and found that the majority of the 37-day difference in mean total time spent in NHS care for failed fixations was due to an increase in acute hospital bed days rather than community hospital days.

#### 3.2.2. Return to Prefracture Residential Status

Several studies have noted a downgrade in patients' discharge destination following the failure of internal fixation compared to that of uncomplicated cases. Eastwood [10] noted that patients requiring revision surgery were 35 times more likely to be referred to continuing care, with a consequent increase in social dependency. Other studies support these findings with patients less likely to return to their own home and more likely to be referred for continuing rehabilitation [9, 11]. It appears, however, that this downgrade of residential status is limited to the short term and several authors have noted no difference at long-term followup [12, 13].

#### 3.2.3. Quality of Life (QoL) and Functional Outcome

While all patients suffer a decrease in their quality of life after hip fracture, this is particularly evident in patients who have a failed fixation. Tidermark et al. [8] noted that mean quality of life (EQ-5D index score) was higher at each follow-up assessment for those with healing fractures than those who suffered a failure of fixation: at 4 months, 0.66 versus 0.49 (*P* < 0.05) and at 17 months, 0.62 versus 0.31 (*P* < 0.005). At inclusion, there had been no difference between the groups. They also noted a more profound decrease in body weight and lean body mass at 6 months in the fixation failure group. Other studies support this additional impact on quality of life in the short term, noting lower QoL scores and increased use of walking aids at 4–6-month followup [13–15]. 

With regard to long-term outcomes, studies reveal somewhat diverse findings. Frihagen et al. [14] noted that patients with failed IF requiring revision to hemiarthroplasty had worse QoL (Hip Score and Visual Analogue Score) at 4 months, but they did not differ from the healed IF group at 1- and 2-year followup. Similarly, Bjørgul and Reikerås [12] observed no difference at long-term followup in either pain scores or in the proportion of patients retaining their ability to be independent outdoor walkers. In contrast to this, however, several other studies have noted significantly impaired functional outcome and quality of life at one-to-five-year followup. Keating et al. [16], Magaziner et al. [17], and Tidermark [8], all observed that functional outcome for patients managed with IF who did not have subsequent surgery was clearly better than those who required revision surgery at 1-year followup, while Nilsson et al. [18], examining quality of life at 5-year followup via the Nottingham Health Profile (NHP Parts I and II), observed that patients with healed fractures had fewer problems with sleep, housework, and hobbies, and thus functioned better than patients who had required a secondary procedure. 

Additionally, it is often assumed that the conversion of a failed IF for either femoral neck or peritrochanteric fractures is a straightforward solution to the problem. Studies, however, that have compared the outcome of primary arthroplasty (either for osteoarthritis or hip fracture) versus revision of failed fixation do not bear this out [14, 19–21]. There is increased surgical difficulty, more complications (with dislocation and infection rates approximately doubled). Functional outcome is also inferior and there is evidence that the survivorship of these revision prostheses is shorter. There are several reasons for this: patients frequently become profoundly disabled after failed IF leding to more muscle wasting and disuse osteoporosis. Previously operated sites may retain inflammatory tissue or act as a nidus for infection; removal of retained hardware can involve extensive dissection and further damage bone structure, while empty screw holes can lead to inadequate cement pressurisation; fracture collapse may also frustrate attempts at restoration of equal leg lengths.

#### 3.2.4. Mortality

As hip fractures—and their surgical treatment—carry a well-documented increased mortality risk, it is of particular interest to examine the effect of additional surgery in the same patient group. While Thakar et al. [9] did note a significant increase in the probability of mortality following re-operation, other studies have observed only transient increases in mortality during either the initial period of hospitalisation [22] or during the first 6 months [23]. Revision procedures after this did not increase mortality risk and at long-term followup, several authors noted no overall difference in mortality between the groups [10–13, 24, 25]. It is likely, however, that patients passed fit to undergo a secondary operation are a subgroup within this population with a bias towards better survival. 

#### 3.2.5. Cost

Table 5 outlines the findings from several studies with regard to the additional cost of fixation failure. While the method of cost assessment and currency differ across studies, a clear trend can be seen with revision surgery for failed IF leading to an overall doubling of costs.

## 4. Discussion

Fragility fractures of the hip, with their well-documented impact on morbidity and mortality, probably represent the most devastating outcome of osteoporosis in the elderly. Further complicating management, however, is that weakened osteoporotic bone and vulnerable vascular supply predispose toward a failure of fixation. As our population becomes increasingly older and the incidence of hip fractures continues to rise, even low failure rates will constitute a major challenge to health care systems. Central then to planning for these changes is an understanding of both the incidence of such failures of fixation and the impact they can have. 

Overall, the incidence of fixation failure in osteoporotic hip fractures ranges from 5% in peritrochanteric fractures to 15% and 41% in undisplaced and displaced fractures of the femoral neck, respectively. Our findings, in general, support the view that failed internal fixation of these fragility fractures carries a poor prognosis: it leads to a twofold increase in the length of hospital stay and a doubling of healthcare costs. Patients are more likely to suffer a downgrade in their residential status upon discharge with a consequent increase in social dependency. Furthermore, the marked disability and reduction in quality of life evident before salvage procedures may persist at long-term followup. The risk, of course, for the elderly patient with a prolonged period of decreased functioning is that the disability becomes permanent. Despite this, however, no clear link between revision surgery and an increase in mortality has been demonstrated in the literature. 

In view of these findings, of particular relevance then is the discussion between IF and arthroplasty in the management of osteoporotic fractures of the hip. While there is a general consensus that elderly patients with an undisplaced (Garden I and II) fracture of the femoral neck can achieve good results after IF with regard to healing, function, and QoL [8, 26], the optimum management of displaced (Garden III and IV) fractures has previously been a source of debate. A recent meta-analysis examining this issue by Parker and Gurusamy [27] noted that although IF was associated with less initial operative trauma when compared to arthroplasty, there was a significantly higher re-operation rate of 40% versus 11%. Definitive conclusions could not be made regarding the length of hospital stay, return to prefracture residential status, or mortality, but pain and functional outcome did appear to be better for those undergoing a cemented arthroplasty in comparison with fixation. Similar findings were noted by Bhandari et al. in 2003 [28] and Rogmark and Johnell in 2006 [29]. The higher re-operation rate associated with IF is also reflected in studies reporting on financial cost which generally show fixation to be the more expensive option; although IF may have the advantage of a lower initial implant cost, this is outweighed by the high costs associated with repeat admissions and revision surgery [16, 30–32]. Reflecting the high failure and re-operation rates observed with IF, the majority of these fractures are managed by arthroplasty in modern orthopaedic practice. For peritrochanteric fractures of the femur, IF remains the mainstay of treatment, current controversy focusing on intra- versus extramedullary devices and cephalic fixation techniques.

In summary, our findings emphasise the importance of optimising patient surgery in order to reduce the incidence of fixation failure and the associated health and social costs. Furthermore, as arthroplasty has become the preferred treatment method for many of these patients, future studies should focus on which arthroplasty would best serve different patient groups.

## Figures and Tables

**Table 1:** Incidence of fixation failure and reoperation in undisplaced fractures of the femoral neck (Garden I and II). All patients >60 years.

Study	Year	Fixation method	Mean age	Followup (months)	*n*	Fixation failure *n* (%)	Revision surgery *n* (%)
Lee et al. [33]	2008	MIDHS, SHS, and CS	72.3	12	90	7 (7.8)	7 (7.8)
Bjørgul and Reikerås [12]	2007	CS	Nr	39	225	37 (16.4)	42 (18.7)
Yih-Shiunn et al. [34]	2007	SHS, CS	71.6	12	84	8 (9.5)	8 (9.5)
Chiu and Lo [35]	1996	Knowles pinning	73	74	250	42 (16.8)	42 (16.8)
Hui et al. [36]	1994	SHS	80	6	57	11 (19.2)	10 (17.5)

Total/average					706	105 (14.8)	109 (15.4)

Nr: not recorded; CS: cannulated screws; MIDHS: minimally invasive dynamic hip screw; SHS: sliding hip screw.

**Table 2:** Incidence of fixation failure and reoperation in displaced fractures of the femoral neck (Garden III and IV). All patients >60 years.

Study	Year	Fixation method	Mean age	Followup (months)	*n*	Fixation failure *n* (%)	Revision surgery *n* (%)
Parker et al. [37]	2010	CS	82.2	180	226	95 (42)	114 (50.4)
Leonardsson et al. [38]	2010	CS, HP	81.5	124	217	94 (43.3)	125 (57.6)
Frihagen et al. [14]	2007	CS	83.2	24	111	46 (41.4)	47 (42.3)
Johansson et al. [39]	2006	CS	84	24	78	37 (47.4)	34 (43.6)
Blomfeldt et al. [19]	2005	CS	81.4	48	53	22 (41.5)	25 (47.2)
Blomfeldt et al. [40]	2005	CS	84	24	30	9 (30)	10 (33.3)
Rödén et al. [41]	2003	von Bahr screws	81	60	53	34 (64.2)	31 (58.5)
Davison et al. [42]	2001	SHS	73	60	93	30 (32.3)	28 (30.1)
Neander et al. [43]	1997	CS	86	18	10	1 (10)	1 (10)
Jónsson et al. [44]	1996	HP	79	24	24	9 (37.5)	7 (29.2)
van Vugt et al. [45]	1993	SHS	75.3	36	21	6 (28.6)	6 (28.6)
Soreide et al. [46]	1979	von Bahr screws	78	12	51	13 (25.5)	11 (21.6)

Total/average					967	396 (41)	439 (45.4)

Nr: not recorded; CS: cannulated screws; HP: hook pin; SHS: sliding hip screw.

**Table 3:** Incidence of fixation failure and reoperation in peritrochanteric fractures of the femur. All patients >60 years.

Study	Year	Fixation method	Mean age	Followup (months)	*n*	Fixation failure *n* (%)	Revision surgery *n* (%)
de Grave* et al. [47]	2012	GN, ACE TN	74.9	12	112	4 (3.6)	4 (3.6)
Stern* et al. [48]	2011	SHS, GN, PFNA, and DHS Blade	86.4	12	269	11 (4.1)	13 (4.8)
Garg^θ^ et al. [49]	2011	SHS, PFNA	62.3	40	81	6 (7.4)	7 (7.4)
Varela-Egocheaga^¥^ et al. [6]	2009	PCP, GN	82	12	80	3 (3.8)	Nr
Pajarinen* et al. [50]	2005	SHS, PFN	81	4	108	4 (3.7)	4 (3.7)
Utrilla* et al. [51]	2005	SHS, TGN	80	12	163	10 (6.2)	5 (3.1)
Papasimos^θ^ et al. [52]	2005	SHS, PFN, and TGN	81.2	12	120	12 (10)	11 (9.2)
Miedel^θ^ et al. [53]	2005	GN, MSP	84	12	217	13 (6)	12 (5.5)
Saudan* et al. [54]	2002	SHS, PFN	83	12	168	4 (2.4)	8 (4.8)
Harrington^θ^ et al. [55]	2002	SHS, IMHS	83	12	102	6 (5.9)	Nr
Sadowski^θ^ et al. [7]	2002	95° DCS, PFN	79	12	35	8 (22.9)	8 (22.9)
Hoffman* and Lynskey [56]	1996	SHS, IMHS	82	3.7	110	4 (3.6)	2 (1.8)
Park* et al. [57]	1998	SHS, GN	73	18.5	60	3 (5)	1 (1.7)
Hardy* et al. [58]	1998	SHS, IMHS	81	12	100	5 (5)	7 (7)
Kukla* et al. [59]	1997	SHS, GN	83	6	89	0 (0)	2 (2.3)
Radford* et al. [60]	1993	SHS, GN	80	12	200	18 (9)	9 (4.5)
Leung* et al. [61]	1992	SHS, GN	80	7	186	13 (7)	6 (3.2)

Total/average					2200	97 (5)	99 (4.9)

Nr: not recorded; GN: Gamma Nail; TN: Trochanteric Nail; SHS: sliding hip screw; PFNA: Proximal Femoral Nail Antirotation; DHS Blade: Dynamic Hip System Blade; PCCP: Percutaneous Compression Plate; PFN: Proximal Femoral Nail; TGN: Trochanteric Gamma Nail; MSP: Medoff Sliding Plate; IMHS: Intramedullary Hip Screw; DCS: Dynamic Condylar Screw.

^¥^stable fractures only; *Stable and unstable fracture patterns; ^θ^unstable fractures only.

**Table 4:** Impact of fixation failure on length of stay (LOS).

Study	Year	LOS uncomplicated cases (days)	LOS revision cases (days)	Fold change
Thakar et al. [9]	2010	30	67.1	↑ 2.2
Foss et al. [24]	2007	12	55	↑ 3.2
Sipilä et al. [13]	2004	8	17	↑ 2.1
Tidermark et al. [62]	2003	17	31	↑ 1.8
Palmer et al. [11]	2000	29.2	54.4	↑ 1.8

Average				↑ 2.2

**Table 5:** Additional cost of fixation failure.

Study	Year	Cost uncomplicated cases	Cost revision cases	Net additional cost	Fold change
Thakar et al. [9]	2010	*£* 8,976	*£* 20,095	*£* 11,119	↑ 2.2
Frihagen et al. [63]	2010	€ 33,301	€ 66,388	€ 33,087	↑ 2
Johansson et al. [39]	2006	€ 9,000	€ 18,300	€ 9,300	↑ 2
Rogmark et al. [30]	2003	$ 12,000	$ 29,000	$ 17,000	↑ 2.4
Palmer et al. [11]	2000	*£* 5,215	*£* 8,676	*£* 3461	↑ 1.7

Average					↑ 2
